# Harnessing *Trichoderma* spp.: A Promising Approach to Control Apple Scab Disease

**DOI:** 10.3390/pathogens13090752

**Published:** 2024-09-02

**Authors:** Safae Gouit, Ismahane Chair, Zineb Belabess, Ikram Legrifi, Khadija Goura, Abdessalem Tahiri, Abderrahim Lazraq, Rachid Lahlali

**Affiliations:** 1 Phytopathology Unit, Department of Plant Protection, Ecole Nationale d’Agriculture de Meknès, Km10, Rte Haj Kaddour, BP S/40, Meknès 50001, Morocco; safae.gouit@gmail.com (S.G.); ichair@enameknes.ac.ma (I.C.); zineb.belabess@inra.ma (Z.B.); ikramlegr@gmail.com (I.L.); k.goura@edu.umi.ac.ma (K.G.); atahiri@enameknes.ac.ma (A.T.); 2 Laboratory of Functional Ecology and Environmental Engineering, Sidi Mohamed Ben Abdellah University, P.O. Box 2202, Route d’Imouzzer, Fez 30000, Morocco; lazraqab@gmail.com; 3 Plant Protection Laboratory, Regional Center of Agricultural Research of Meknès, National Institute of Agricultural Research, Km 13, Rte Haj Kaddour, BP 578, Meknès 50001, Morocco; 4 Laboratory of Biotechnology and Valorization of Phyto-Resources, Faculty of Sciences, Moulay Ismail University of Meknes, Avenue Zitoune, Meknès 50000, Morocco

**Keywords:** apple scab, biocontrol, *V. inaequalis*, *Trichoderma*, mode of action

## Abstract

Apple scab, caused by the pathogenic fungus *Venturia inaequalis*, can result in significant economic losses. The frequent use of fungicidal products has led to the emergence of isolates resistant to commonly used active substances. Therefore, biological control offers a sustainable alternative for managing apple scab. In this study, eight *Trichoderma* isolates were evaluated against five different isolates of *V. inaequalis* isolated from the Fes-Meknes region. The biocontrol potential of these *Trichoderma* isolates had previously been demonstrated against other pathogens. The results indicated that the inhibition rate of mycelial growth of *V. inaequalis* obtained with *Trichoderma* spp. isolates ranged from 50% to 81%, with significant differences observed among the pathogenic isolates after 5 and 12 days of incubation. In addition, the in vitro tests with *Trichoderma* cell-free filtrates showed inhibition rates ranging from 2% to 79%, while inhibition rates ranged from 5% to 78% for volatile compound tests. Interestingly, the inhibition of spore germination and elongation was approximately 40–50%, suggesting the involvement of antifungal metabolites in their biocontrol activities. The in vivo bioassay on detached apple leaves confirmed the biocontrol potential of these *Trichoderma* isolates and demonstrated their ability to preventively control apple scab disease. However, their efficacies were still lower than those of the fungicidal product difenoconazole. These findings could contribute to the development of an effective biofungicide based on these *Trichoderma* isolates for reliable and efficient apple scab control.

## 1. Introduction

In Morocco, apple orchards represent a major crop, occupying approximately 51.871 hectares, after almond trees [1]. Apple production occupies 20% of the area cultivated with rosaceous fruit trees [2], thus constituting the backbone of the economy in these production areas. However, apples are subject to several biotic and abiotic factors, which cause significant economic losses to commercial fruit growers. Among the common pests and diseases affecting cultivated apples (*Malus* × *domestica*), apple scab caused by *Venturia inaequalis* Cooke (Winter) stands out as the most devastating disease, causing significant economic losses in terms of fruit quality and yield. Without sufficient disease control measures, economic losses can escalate by up to 70% of the production value, potentially affecting 100% of the yield [3].

The ascomycete fungus *Venturia* is a hemibiotrophic pathogen that involves both asexual and sexual phases, adapting to seasonal changes for survival and propagation. The optimal time for it to infect apple trees is the spring season, when ejected ascospores germinate and form mycelium. During the summer, this mycelium propagates infections through its asexual cycle. In autumn, the fungus enters its sexual reproductive cycle to enhance its survival through the winter [4]. Managing apple scab heavily depends on fungicides, with sprays routinely applied every 7 to 10 days from bud burst until the risk of scab subsidies [5]. The control often requires more than 12 applications in a single growing season [6,7]. However, such frequent applications escalate input costs, diminish fungicide efficacy, and increase resistance cases. Additionally, they pose significant risks to human health and can cause environmental degradation. Therefore, developing alternative control methods and eco-friendly solutions is essential. To address these challenges, several studies have explored the use of biological control products to combat resistance while minimizing risks to human health and the environment [8].

According to Baker [9], the term ‘biocontrol’ or ‘biological control’ was first introduced by Tubeuf in 1914 and later by Smith et al. [10], focusing on plant pathogens and insects, respectively. Biocontrol involves reducing plant pest populations through naturally occurring organisms, which are part of integrated disease management. A diverse array of biocontrol agents or bio-fungicides exist in the ecosystem and need to be isolated for practical use [11]. These agents offer advantages such as low production costs, long-lasting effects on pathogen growth, and no adverse impact on human health [12]. Indeed, biological control can involve using living organisms (such as fungi, bacteria, and viruses) or their products and metabolites. Among the various species employed as biocontrol agents, *Trichoderma* is widely used against different types of plant pathogens [12].

*Trichoderma* spp. are filamentous fungi found in all types of agricultural soils and decaying wood. These fungi exhibit parasitic behaviour toward many soil-borne and foliar plant pathogens; in addition to its ability to control plant diseases, *Trichoderma* spp. also promote plant growth and development, enhancing plant resistance and leading to increased crop production [13]. Only a few studies have highlighted the effects of *Trichoderma* species in controlling apple scab. In a study by Muresan [14], researchers collected 60 endophytic fungal isolates from apple trees. Notably, *Penicillium* and *Trichoderma* species were predominant, making up 60% of the isolates. Of these, 55 isolates demonstrated significant in vitro inhibition of *V. inaequalis*. Their antagonistic activity involves mycoparasitism, antibiotic production, nutrient competition, and the induction of systemic resistance in plants. These characteristics make these fungi a valuable component in integrated crop management practices, contributing to more sustainable and environmentally friendly agricultural systems [12]. The main objective of our study was to assess the antagonistic effects of eight local *Trichoderma* isolates on the in vitro mycelial growth and conidial germination of five pathogenic strains of *V. inaequalis*. Furthermore, our study aimed to evaluate the in vivo antagonistic activity of two *Trichoderma* isolates that demonstrated the most significant in vitro effects.

## 2. Materials and Methods

### 2.1. Pathogenic Strains

The five strains of *V. inaequalis* used in this study were obtained from the PhytoENA-Meknès Microorganisms Collection (CMENA, Meknes, Morocco). These strains were isolated from symptomatic apple leaves collected from various orchards of apple trees in the Fes-Meknes regions in 2022: ViIF01 (Ifrane), ViAZ, ViIM (Meknès), ViHA (El Hajeb), ViEN01 (ENA-Meknès). The fungal colonies used for various tests were obtained through subculturing and were grown on Potato Dextrose Agar (PDA, Biokar Diagnostics, Zac de Ther, France) at a temperature of 25 °C in darkness. The spore suspension of the five isolates was prepared by gently scraping the colonies with a sterile scalpel or a sterile lancet needle. To recover the spores, this culture was mixed with 5 mL of Sterile Distilled Water (SDW) containing 0.05% of Tween 20. Subsequently, the contents were filtered through Whatman paper to remove debris and mycelium, keeping only the spores. A volume of 10 μL of the spore suspension was then spread on a Bürker counting cell (Roche, Meylan, France), and the concentration was adjusted to 1 × 10^6^ spores/mL [15].

### 2.2. Trichoderma Isolates

*Trichoderma* isolates were obtained from the PhytoENA-Meknès Microorganisms Collection (CMENA). Eight *Trichoderma* isolates were isolated from the rhizosphere of various horticultural crops, including grapevine, apple, olive, and citrus trees, during a survey conducted in 2021. The fungal cultures were maintained on PDA medium for 5 days in a dark incubator at 25 °C. These fungi isolates included *Trichoderma virens* (Ol4), *Trichoderma atroviride* (Y28), other isolates of *Trichoderma* spp. (X31 Tricho, G, and Nef9)), *Trichoderma atroviride* (T2, PQ240702), and *Trichoderma harzianum* (T1, PQ240703). The GenBank numbers PQ240702 and PQ240703 correspond to sequences of the Internal Transcribed Spacer (ITS) region. The fungal suspension was prepared from a 7-day culture grown on PDA medium at 25 °C and was then adjusted to a concentration of 1 × 10^6^ spores/mL using a Bürker cell.

### 2.3. Dual Culture Bioassay

A dual culture plate test was conducted to assess the ability of *Trichoderma* isolates to inhibit *V. inaequalis* growth. In this test, 5 mm agar plugs, cut with a sterile scalpel from 7-day-old *Trichoderma* colonies and 12-day-old *V. inaequalis* colonies were placed opposite each other at the edges of a 9 cm Petri dish containing PDA. Five replicate plates were used for each *Trichoderma* isolate as well as the control (*V. inaequalis* grown alone). The paired cultures were incubated at 25 °C and were examined when mycelium filled the control plates according to Elshahawy and El-Mohamedy [16]. The reduction in *V. inaequalis* mycelial growth was calculated using the following formula:

Growth reduction (%) = [(C − T)/C] × 100,

where C = average growth area (cm^2^) of *V. inaequalis* in the control and T = average growth area (cm^2^) of *V. inaequalis* in the presence of the *Trichoderma* isolates

### 2.4. Inhibitory Effect of Volatile Organic Compounds (VOCs)of Trichoderma Isolates

The effect of volatile metabolites produced by *Trichoderma* isolates on *V. inaequalis* was assessed using the method described by Raut et al. [17]. Petri dishes containing PDA were individually inoculated with a mycelial plug of *V. inaequalis* and a mycelial plug of *Trichoderma* isolates. The bottoms of the dishes were then sealed with four layers of Parafilm, with one base placed over the other. Control sets did not include the antagonist. The cultures were incubated at 25 °C, and the radial growth of *V. inaequalis* was measured at 5- and 12-days post-incubation. The inhibition rate was calculated using the following formula:

Inhibition rate = [(D1 − D2)/D1] × 100,

where D1 = diameter of radial growth of *V. inaequalis* in the control and D2 = diameter of radial growth of *V. inaequalis* in the treatment.

### 2.5. Inhibitory Effect of Trichoderma Isolate-Free Cell Filtrates on V. inaequalis Mycelial Growth

Free cell filtrates of *Trichoderma* isolates were prepared by growing the isolates in 100 mL of Potato Dextrose Broth (PDB, Biokar Diagnostics, Zac de Ther, France), shaken on a rotary shaker at 130 rpm for 72 hrs at room temperature. The broth was then centrifuged at 4800 rpm for 35 min. The supernatant was filtered through Whatman No. 1 filter paper, and the resulting sterilized liquid was used for further studies [16]. The supernatant (22.5 mL) was re-filtered through a sterile 0.22 μm Millipore filter (Merck Millipore, Darmstadt, Germany), directly added to 227.5 mL molten sterilized PDA to obtain a 9% concentration immediately before solidification, and then poured into Petri dishes. PDA supplemented with SDW served as a control. A mycelial plug of *V. inaequalis* was placed at the centre of both the test and control plates and incubated at 25 °C for 12 days. The linear growth of the mycelium was measured, and the percentage of inhibition was calculated using the specified equation. All experiments were repeated twice with three replicates.

### 2.6. Inhibitory Effect of Spore Germination

The inhibitory effect of *Trichoderma* isolates on the germination of *V. inaequalis* spores was assessed using a modified protocol from Elshahawy and El-Mohamedy [16], adapted for *V. inaequalis* spore production. Equal volumes of *V. inaequalis* spores and *Trichoderma* conidia (both at a concentration of 1 × 10^3^/mL) were mixed. This mixture was incubated at 25 °C for 24 h. Samples were then observed under a light microscope to determine the percentage of ungerminated spores, with 100 spores counted per treatment. A spore was considered germinated if the length of the germ tube was equal to or greater than the length of the spore [18]. Results are presented as the inhibition rate of spore germination, calculated as described previously [18] using the following formula:

GI (%) = (Gc − Gt/Gc) ×100,

where Gc and Gt represent the average number of spores germinated in controls and treatments, respectively.

Five spores that had germinated were selected for the measurement of their germ tube length. The enumeration was carried out diagonally using a Bürker cell and a digital counter. As for the measurement of germ tube length, we utilized software called ImageFocus Plus V2 on our laptop, which was connected to the light microscope (Ceti Microscopes NLCD-307B, Chalgrove, UK).

### 2.7. Effect of In Vivo Antagonists on V. inaequalis on Detached Leaves

Adapting the protocol of Nicholson et al. [19], with modifications. Detached apple leaves were immediately immersed in Sterile Distilled Water (SDW). After gently rubbing to remove hairs and debris, they were rinsed with SDW for several minutes. The petiole closest to the base of each leaf was excised, and the leaves were then rinsed three additional times with SDW. Each leaf was placed adaxial side up on sterilized paper inside a 150 mm diameter Petri dish, ensuring good contact between the cut petiole and the SDW, following the method described by Yepes and Aldwinckle [20].

For the preventive treatment, four-day-old *Trichoderma* spp. cultures were harvested and resuspended in a sterile PDB solution supplemented with 0.01% Tween 20, adjusted to a concentration of 1 × 10^6^ spores/mL). Each leaf was sprayed with approximately 5 mL of the suspension using a handheld sprayer. The leaves were allowed to air dry for approximately 2 h before being inoculated with a conidial suspension of *V. inaequalis* (1 × 10^6^ spores/mL) [19]. Petri dishes were then sealed with Parafilm and incubated in a growth chamber for 30 days, under the same conditions used for maintaining the pathogens. The fungicide difenoconazole (Dif, Score®250 EC, Syngenta, Morocco) was used at a concentration of 1 ppm, prepared from a stock solution of 250 g/L. The fungicide was applied 2 h before inoculation with a conidial suspension of *V. inaequalis* (1 × 10^6^ spore/mL) [19].

For the curative treatment, the upper surface of the leaves (after disinfection) was kept dry in Petri dishes placed in a laminar flow hood before applying the spore suspension of *V. inaequalis* at a concentration of 1 × 10^6^ spores/mL. Both *Trichoderma* spp. isolates (Ol4 and Y28) were applied at the same concentration and the fungicidal treatment (Difenoconazole) was applied at the same concentration as the preventive treatment. Visual assessment of infected leaf surfaces was conducted using a scoring scale developed by Calenge et al. [21], assigning seven categories based on the percentage of leaf disease development: (0) no visible lesions, (1) <1% of leaf surface with lesions, (2) 1–5%, (3) 5–10%, (4) 10–25%, (5) 25–50%, (6) 50–75%, and (7) 75–100%.

### 2.8. Statistical Analyses

Statistical analyses were performed using SPSS statistical software (version 20). To check the effect of treatment, Analysis of Variance (ANOVA) was applied for each trial and for each pathogen separately. When the effect was revealed to be significant, a Student–Newman–Keuls (SNK) test was performed for means separation at a significance level of 0.05. For the in vitro assays, the data were analysed by 3 ANOVA factors: (1) 9 treatments (8 *Trichoderma* spp. + 1 fungicide/fungus alone without treatment), (2) 2 incubation times (after 5 days and after 12 days of incubation), and (3) the five pathogenic strains (ViAZ, ViIM, ViHA, ViIF01, and ViEN01). This trial was repeated twice with 4 replicates. For the in vivo disease severity assay on detached leaves treated with antagonists, disease severity was calculated after four weeks of fungal inoculation. Data were analysed by 3 ANOVA factors: (1) 5 treatments (2 *Trichoderma* spp. to be tested “Ol4; Y28” + 1 fungicide/fungus alone without treatment), (2) treatment (preventive, curative), and (3) the five pathogenic strains (ViAZ, ViIM, ViHA, ViIF, and ViEN) as fixed factors and severity as the response. This trial was repeated twice with 4 replicates.

## 3. Results

### 3.1. In Vitro Effect on Mycelial Growth

**Figure 1:**
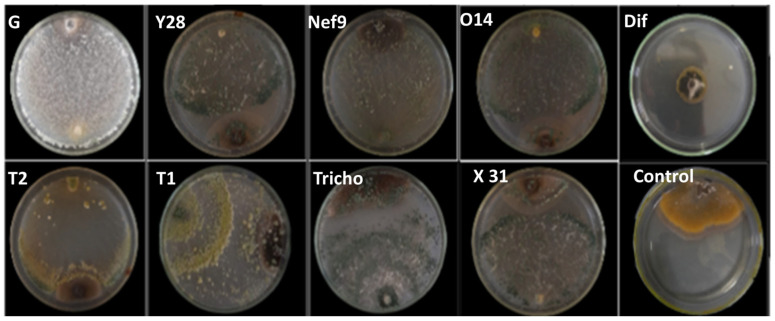
In vitro dual culture bioassay of *Trichoderma* spp., fungicidal product, and *V. inaequalis* strain ViAZ after 12 days of incubation at 25 °C.

After 5 days of incubation, the results showed that the inhibition rate of mycelial growth varied depending on the applied treatments (Table 1 and Figure 1). For the ViAZ strain, four *Trichoderma* isolates (Y28, G, Ol4, and X31) achieved inhibition rates exceeding 50%, while the remaining isolates exhibited inhibition over 40%. The synthetic fungicide proved to be the most effective, with an inhibition rate of 69.80%. However, most *Trichoderma* isolates exhibited inhibition rates below 50% against ViIM, except for Ol4 and Y28, which achieved inhibition rates of 56.60% and 52.55%, respectively. For the ViIF01 strain, the Ol4 strain exhibited a higher inhibition rate (73.82%) than the fungicide (69.37%), while the Y28 strain showed an inhibition of 69.48%, comparable to that of the fungicide. The other isolates demonstrated inhibition rates ranging from 55.51% (X31) to 38.50% (G). Against ViHA, only the Ol4 strain achieved inhibition exceeding 60%, although this was still lower than the fungicide’s inhibition rate of 80.23%. The remaining isolates showed inhibition rates between 39.75% and 45.35%. For the ViEN01 strain, four isolates (G, Tricho, Y28, and Ol4) exhibited inhibition rates exceeding 50%, while the rest showed inhibition rates above 40%. All these rates were lower than that of the fungicide, which had an inhibition rate of 71.64%.

The in vitro effect of *Trichoderma* species on the mycelial growth of *V. inaequalis* after 12 days of incubation is depicted in Figure 1 and Table 2. Isolates T1 and Y28 exhibited significantly high inhibition against ViAZ, surpassing 70%. Other *Trichoderma* isolates showed inhibition rates exceeding 60%. Against the ViIM strain, isolates Ol4, Nef 9, X31, and G displayed inhibition rates above 60%. Notably, strain X31 was highly effective against the ViIF01 strain, with an inhibition rate exceeding 80%, while isolates Ol4, Y28, and Tricho all surpassed 70%. Certain *Trichoderma* isolates exhibited effects above 60%. Specifically, only isolates T2 and Tricho showed inhibition rates over 60% against the ViHA strain. Other isolates had inhibition rates exceeding 50%. Against ViEN01, all isolates except Tricho and X31 demonstrated inhibition rates over 60%, with Tricho and X31 showing rates of 50.19% and 58.24%, respectively. The fungicide achieved the highest rate at 75.63%.

According to the results presented in Table 1 and Table 2, the inhibitory capacity of all tested *Trichoderma* spp. strains significantly improved compared to a 5-day incubation period, with the exception of strain Ol4 against the pathogenic strains ViHA and ViEN01, as well as strain Tricho against ViEN01. Additionally, strain Y28 exhibited stability in inhibiting ViEN01.

### 3.2. Effect of Volatile Organic Compounds (VOCs)

After a 5-day incubation period, the results showed diverse levels of mycelial growth inhibition among different treatments (Table 3). Two isolates, Y28 (69.80%) and X31 (66.52%), demonstrated significant inhibition against ViAZ, both surpassing 60%. In contrast, other isolates exhibited inhibition levels below 40%, with the T1 strain showing the lowest inhibition. Y28 and X31 were the only isolate to achieve inhibition rates exceeding 50% against ViIM, with other isolates showing notably lower inhibition levels, all below 22%. Also, four isolates displayed inhibition rates surpassing 50% against ViIF, namely G (52.07%), X31 (53.46%), Tricho (58.41%), and Y28 (59.44%), while Nef9 exhibited the lowest inhibition at 16.25%. Against ViHA, three isolates exceeded 60% inhibition, with strain T1 having the lowest inhibition rate at 13.79%. For ViEN01, three isolates (Tricho, X31, and Y28) showed inhibition rates greater than 50%, while the rest displayed rates ranging from 32.88% to 11.61%.

**Figure 2:**
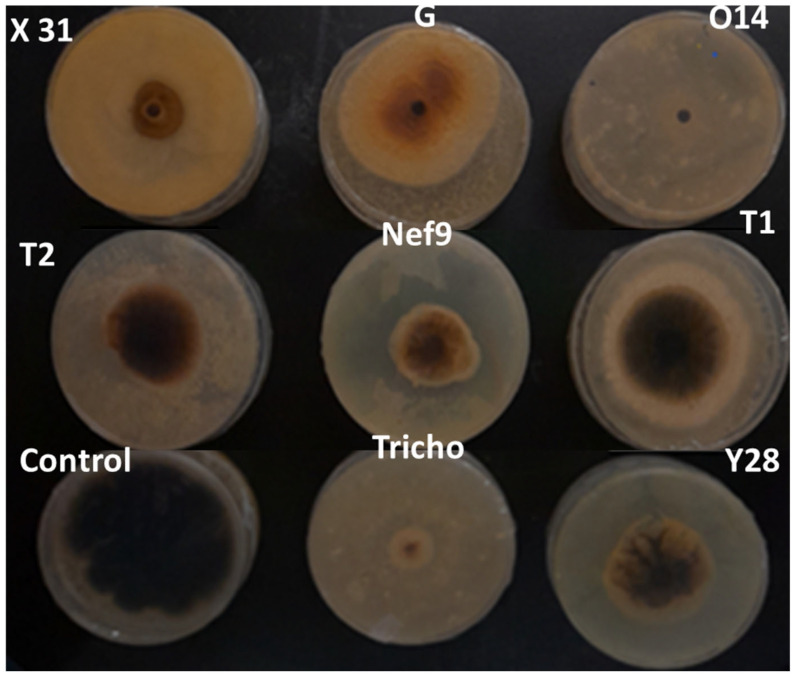
Distance dual confrontation VOC bioassay between *Trichoderma* spp. and *V. inaequalis* strain ViAZ after 12 days of incubation at 25 °C.

The in vitro effect of *Trichoderma* spp. on the mycelial growth of *V. inaequalis* after 12 days of incubation is presented in Figure 2 and Table 4. Isolates Y28 and X31 demonstrated mycelial growth inhibition rates exceeding 60% against ViAZ, while the other isolates did not exceed 47%, with T1 and T2 showing the lowest rates. For ViIM, Y28 and X31 also exhibited the highest inhibition rates (exceeding 60%), while the others did not surpass 40%. Against ViIF, three isolates (Tricho, X31, and Y28) displayed inhibition rates above 50%, whereas the remaining isolates exhibited considerably lower rates. For ViHA, Tricho, X31, and Y28 surpassed 70% inhibition, while Nef9 was the only other isolate to exceed 50%. The remaining isolates varied between 37.89% and 14.01%. Against ViEN, Tricho (58.65%), X31 (64.79%), and Y28 (71.34%) showed inhibition rates exceeding 50%, while the others ranged from 48.22% to 26.02%

According to the results presented in Table 3 and Table 4, the inhibitory capacity of all tested *Trichoderma* spp. significantly improved compared to a 5-day incubation, with the exception of isolate T2 against ViAZ, and isolates G, Ol4, T1, and X31 against ViHA. Additionally, isolate T2 displayed reduced inhibitory capacity against ViIF01, and strain T1 showed lower inhibition against ViIM.

### 3.3. In Vitro Effect of Trichoderma- Free Cell Filtrates on Mycelial Growth of V. inaequalis

The analysis after 5 days of incubation revealed variations in mycelial growth inhibition (Table 5), using *Trichoderma* spp. free cell filtrates at a 9% concentration. Isolates G, T1, and Tricho exhibited inhibition levels surpassing 50% against ViAZ, while Y28 had the lowest inhibition rate at 16.25%. For ViIM, only one *Trichoderma* isolate showed a high inhibition rate, reaching 63.41%, while the others did not surpass 45%. Against ViIF01, strains T1 (72.60%), Y28 (78.71%), X31 (66.87%), and Nef9 (58.42%) demonstrated inhibitory effect of over 58%, while X31 exhibited the lowest rate at just 24% against ViHA. The strains Nef9 and Y28 achieved inhibition rates exceeding 50%, while X31 showed the lowest rate at 15.94%.

After 12 days of incubation (Table 6), the three *Trichoderma* isolates, G, T1, and X31, exhibited inhibition rates exceeding 50% against ViAZ, while the remaining strains registered inhibition percentages above 40%. Isolate Y28 showed the highest inhibition rate against ViIM at 78.45%, followed by Ol4 with 61.36%. Similarly, Y28 also had the highest inhibition rate against ViIF01 at 78.45%, followed by T1 and Ol4 with respective rates of 56.23% and 50.15%. Notably, Y28 was the only isolate to surpass 60% inhibition against ViHA, while Nef9 and T1 reached 50%. For ViEN01, Y28 again led with an inhibition rate exceeding 70%, followed by OL4 at 62%. The lowest inhibition rate was recorded for X31.

The results from Table 5 and Table 6 demonstrate a significant improvement in the inhibition of the tested *Trichoderma* spp. compared to a 5-day incubation. However, exceptions are observed, particularly with the Tricho and Nef 9 isolates against ViAZ, as well as the G, Nef9, T1, T2, and Tricho isolates for ViEN01, ViHA, ViIF01, and ViIM.

### 3.4. Effect of Filtrates on V. inaequalis Spore Germination

**Figure 3:**
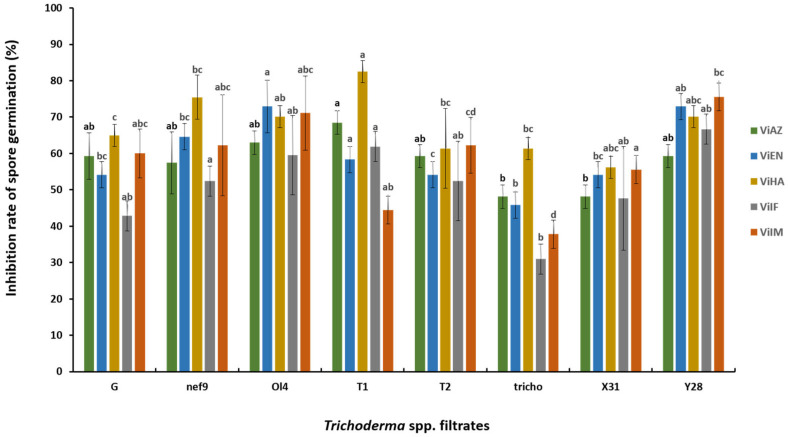
Inhibition rate of spore germination (%) of *V. inaequalis* on a total of 100 spores after 24 h of incubation by the filtrates of antagonistic Trichoderma spp. of concentration 100%. The data representing the mean inhibition rate with the same letter are not significantly different according to the SNK test performed on mycelial growth (*p* < 0.05).

The filtrates of *Trichoderma* spp. isolates at a 100% concentration effectively inhibited spore germination of all five *V. inaequalis* strains (Figure 3). Among the *Trichoderma* isolates, Ol4 and T1 achieved inhibition rates exceeding 60% against VIAZ, while Tricho and X31 had the lowest inhibition rates at 48.15%. For VIEN01, Ol4 and Y28 demonstrated inhibition rates above 70%, with other strains showing rates over 50%, except for Tricho, which had a rate of 45.83%. The highest inhibition rate for ViHA was observed with T1 at 82.46%, followed by Nef9 at 75.44% and Ol4 and Y28 at 70.18%. The lowest inhibition rate was recorded by the G strain at 56.14%. For ViIF01, Y28 achieved the highest inhibition rate at 66.67%, followed by T1 at 61.9%, while Tricho had the lowest rate at 30.95%. Against ViIM, Ol4 (71.11%) and Y28 (75.56%) exhibited inhibition rates above 70%, with Tricho showing the lowest rate at 37.78%

### 3.5. Effect of Filtrates on the Length of the Germ Tube of V. inaequalis

**Figure 4:**
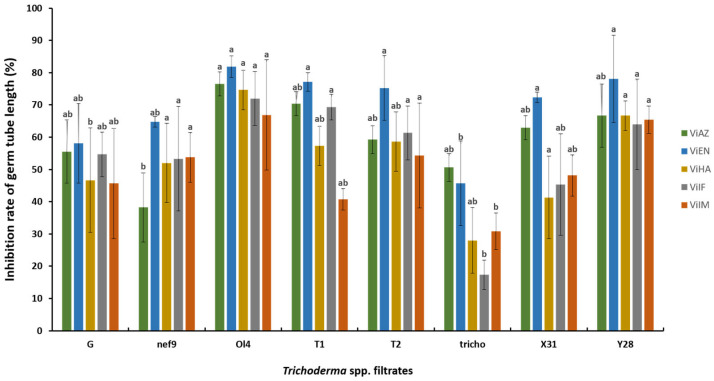
Inhibition rate of germ tube length (%) of *V. inaequalis* after 24 h of incubation by the filtrates of Trichoderma antagonists of concentration 100%. The data representing the mean inhibition rate, with the same letter are not significantly different according to the SNK test performed on mycelial growth (*p* < 0.05).

The cell-free filtrates of *Trichoderma* spp. isolates at a 100% concentration effectively inhibited the elongation of germinated tubes in all five strains of *V. inaequalis* (Figure 4). Results indicated that isolates Ol4 (76.54%) and T1 (70.37%) exhibited inhibition exceeding 70% against ViAZ. In contrast, Nef 9 showed the lowest inhibition rate (38.27%), with germination tube elongation reduced from 170 µm to approximately 0.007 µm. For ViEN01, isolate Ol4 (81.9%) displayed very high inhibition, exceeding 80%. Three other isolates, Y28, T1, and T2, along with X31, showed inhibition rates exceeding 70%. These strains reduced tube elongation to approximately 60 µm and 80 µm, respectively, from an initial length of 350 µm. Isolate Tricho recorded the lowest inhibition rate at 45.71%, with tube elongation of 200 µm. Against ViHA, only isolate Ol4 exhibited efficacy reaching 74.67%, while other strains displayed values exceeding 40%, except for Tricho (28%). Similarly, for ViIF01, Ol4 recorded 72% inhibition, while Tricho displayed an inhibition of 17.33%. Two isolates, Y28 and Ol4, demonstrated inhibition exceeding 60% against ViIM, while the other strains exhibited rates exceeding 40%, except for Tricho (30.86%).

### 3.6. The In Vivo Effect of Antagonistic Trichoderma on Detached Apple Leaves

**Figure 5:**
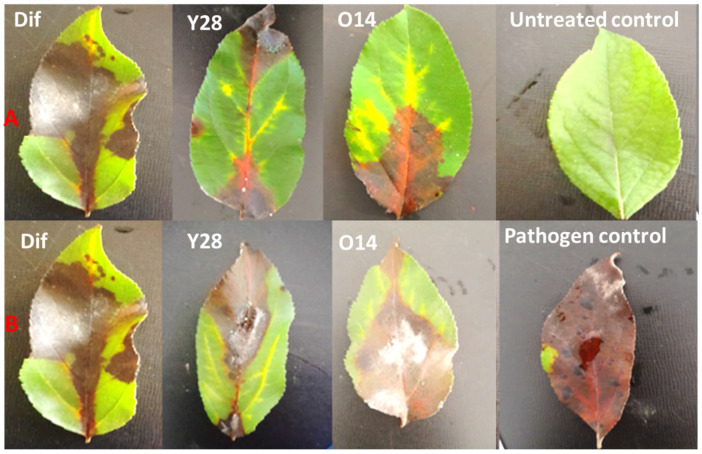
Symptoms of apple scab caused by *V. inaequalis* strain ViAZ observed on detached apple leaves 5 days after preventive (**A**) and curative (**B**) treatments with fungal antagonists *T. atroviride* (Y28), *T. virens* (O14), and the fungicidal product (Dif). Detached leaves treated with water and the pathogen served as untreated and treated controls.

The results of in vivo tests showed that disease severity varied among isolates, depending on the fungal isolate and fungicidal product applied. Significant differences were observed in the severity of apple scab on detached leaves treated with three treatments (antagonists Y28, O14, and fungicide), in both preventive and curative approaches (Figure 5 and Table 7). Indeed, antagonistic *Trichoderma* species and the fungicide applied in curative treatments demonstrated significantly higher severity than in preventive treatments. Furthermore, the efficacy of curative treatments was higher when applied after 5 days compared to application after the onset of symptoms.

The results showed that mean disease severity varied depending on the treatments used. When difenoconazole was applied, the severity was significantly reduced compared to *Trichoderma*-based treatments. However, in the curative treatments, the severity was comparable between the antagonist and the fungicide for the ViAZ and ViIF01 strains. The mean severity of the control for the ViHA isolate was slightly higher than for the other isolates, namely ViIF01, ViAZ, ViEN01, and ViIM, with percentages of 98.50, 97.50, 97, 98.25, and 96.75, respectively. For preventive treatment, the severity ranged from 41.25% to 97% for the ViAZ, ViEN01, ViHA, ViIF01, and ViIM isolates, respectively. The fungicide application significantly reduced severity, especially for the ViEN01 isolate, with percentages of 58.75% after 5 days and 60.25% after the onset of symptoms when curative treatment was applied (Table 7).

For the *Trichoderma* spp. Y28 and Ol4 isolates, there was a significant reduction in severity in preventive treatment with the ViAZ isolate, reaching 48.50% and 59%, respectively. Similarly, in curative treatment after 5 days, they presented a significant reduction in severity with the ViEN01 isolate, reaching 55% and 61.50%, respectively. Furthermore, with the ViHA and ViIF01 isolates, they showed severity reductions of 74.50% and 76.75%, respectively, after the onset of symptoms.

The results indicate that isolate Y28 recorded lower severity than isolate Ol4 in both treatment types. Furthermore, strain Y28 showed similar efficacy to the fungicide with the ViHA, ViAZ, and ViEN01 isolates, both in preventive treatment and in curative treatment after 5 days (Table 7).

## 4. Discussion

Biological control is an excellent and effective alternative for managing plant diseases [22]. *Trichoderma* spp. represent a promising source of biological control agents. These microorganisms are adapted to coexist with the host plant, presenting minimal adverse effects. They contribute to plant health by generating protective metabolites, inducing resistance to biotic and abiotic stresses, and promoting growth through the production of phytohormones [23,24]. Various studies conducted globally have demonstrated the biological potential of different *Trichoderma* species against fungal pathogens [25]. For instance, Nourian et al. [26] assessed the antifungal properties of these antagonistic agents against *Diplodia bulgarica* in vitro, finding that *Trichoderma zelobreve* inhibited the growth of the pathogen’s mycelia. Similarly, Taha et al. [27] reported that various *Trichoderma* isolates markedly hindered the growth of *Fusarium* species in vitro. The effectiveness of biological control mechanisms is influenced by several factors, including the specific antagonist strain used, the nature of the pathogenic fungus, the characteristics of the host plant, and prevailing environmental conditions [28,29]. In this study, different isolates of *Trichoderma* spp. demonstrated variable in vitro inhibition capacities, due to their intrinsic properties and the effectiveness of the mechanisms of action involved, namely, antibiosis by the secretion of antifungal metabolites and Volatile Organic Compounds (VOCs), as well as parasitism by the secretion of lytic enzymes.

The results of the in vitro direct confrontation test clearly demonstrated the strong inhibitory activity on the mycelial growth of the five pathogenic isolates of *V. inaequalis*. This activity varied depending on the antagonistic *Trichoderma* isolates used and the incubation time. The inhibition rates ranged as follows: 63% to 74% for isolate ViAZ, 53% to 62% for ViIM, 62% to 81% for ViIF01, 55% to 61% for ViHA, and 50% to 68% for ViEN01. More specifically, *T. virens* (Ol4) and *T. atroviride* (Y28) generated the highest inhibition percentages among all *Trichoderma* isolates tested against the five strains of *V. inaequalis*. In the same regard, Muresan [14] demonstrated that the inhibitory effect of *T. asperellum* isolate (FRV21) was greater than 76% against three isolates of *V. inaequalis.* Also, Jimenez el al. [30] demonstrated, through the in vitro double culture assay, that four identified isolates of *Trichoderma* (*T. harzianum*, *T. yunnanense*, *T. lignorum*, and *T. asperellum*) exhibited antagonistic and hyperparasitic activity on the mycelial growth of *V. inaequalis* in just two days.

Moreover, our results align with those of Doolotkeldieva and Bobusheva [31], who demonstrated a positive inhibition of apple scab by *T. viride*, both in vitro and in the field. In various scenarios, *T. virens* has demonstrated broad-spectrum antifungal properties and can act parasitically against certain harmful fungi. Studies have shown that its efficacy is largely due to its rapid growth and potent amylase activity, enabling it to effectively compete for ecological niches [32,33]. The rapid growth of *T. virens* significantly enhances its mycoparasitic capabilities, allowing it to quickly colonize and outcompete pathogenic fungi for resources and space. The strong amylase activity of *T. virens* further degrades the cell walls of the pathogens, leading to their gradual weakening and eventual death. As a result, the pathogens experience slowed growth, structural dissolution, and ultimately, a significant reduction in their population, contributing to more effective biological control [34].

In addition to mycoparasitism and antibiosis, other biocontrol mechanisms for Biological Control Agents (BCAs) in natural conditions have not been extensively studied. Competition for nutrients and space is believed to play a crucial role in the effectiveness of *Trichoderma* species. This form of competition has been observed in other pathosystems. For example, *V. inaequalis* increases the permeability of host plant cell membranes, leading to a greater nutrient supply from the host [35]. While competition for space or nutrients is considered one of the classical mechanisms of biocontrol by *Trichoderma* spp., demonstrating it experimentally is challenging [36]. It is likely that multiple mechanisms are involved in biocontrol systems, though typically only a subset of these mechanisms has been fully elucidated [37].

The results of the volatile compounds revealed that the highest inhibition rates were obtained with the *T. atroviride* strain (Y28) and *Trichoderma* spp. (Tricho and X31). Similar conclusions were reached in the study of Rao et al. [38], where the volatile organic compounds produced by *T. atroviride* strongly inhibited the mycelial growth of *Fusarium* and showed great potential for the root growth of tomato plants. Jeyaseelan et al. [39] also reported that many *Trichoderma* spp. strains that produce volatile metabolites can negatively affect the growth of various phytopathogenic fungi. Previous work by Yu et al. [40] and Strobel [41] have proven the ability of *Trichoderma* species to produce a range of secondary metabolites, including Volatile Organic Compounds (VOCs), as well as different enzymes. In addition, Speckbacher et al. [42] found that certain *Trichoderma* species, such as *T. atroviride, T. gamsii,* and *T. harzianum,* produce the compound 6-pentyl-2H-pyran-2-one (6-PP). In contrast, species like *T. virens* and *T. reesei* do not produce this compound [43,44]. The results indicate that inhibition rates were lower in volatile tests compared to non-volatile tests. This is consistent with findings by Elshahawy and El-Mohamedy [16], who observed that although volatile metabolites from *Trichoderma* spp. exhibit inhibitory effects on pathogens, their effectiveness is not as strong as that of non-volatile metabolites.

The in vivo test results revealed that disease severity was significantly reduced when using *T. atroviride* (Y28) isolates compared to *T. virens* isolates (Ol4). These findings align with those of Jimenez et al. [30], who noted in their in vivo evaluations that applying various *Trichoderma* strains effectively reduced the severity of scab on apple leaves. These effects could be attributed to the mechanisms of action mentioned in the in vitro tests, causing structural changes at the cellular level, disintegration of the cytoplasm, and cell lysis of the pathogenic fungus [36,45]. Indeed, our results are consistent with the observations of Doolotkeldieva and Bobusheva [31], who evaluated the application of *T. viride* on *V. inaequalis* in apple seedlings. They found that after 35 days and two treatments, disease progression in the leaves was stopped, likely due to the reinforcement of the leaf cell wall and the alteration of subcuticular pH, both influenced by secondary metabolites produced by the antagonists. These metabolites are believed to play a crucial role in modulating cellular resistance during leaf development.

Also, it was demonstrated that the highest severity was observed for *T. atroviride* (Y28) in curative treatment, reaching 84.25% against the ViIF01 isolate, while the lowest severity was recorded during the preventive application of the same strain against the ViAZ isolate, at 48.50%. This highlights the importance of timing in the application of antagonistic agents to effectively inhibit disease spread on leaves. Based on these findings, it appears that the effectiveness of *Trichoderma* spp. may involve a direct interaction with the pathogen. This interaction could disrupt the pathogen’s ability to infect or damage the plant, thereby enhancing the effectiveness of the biological control strategy. Further studies could help elucidate the specific nature of this direct interaction and its implications for improving disease management.

Investigating the interactions between the antagonist, the host plant, and the pathogen could reveal valuable insights into the mechanisms of biological control for plant diseases. By understanding how these components influence each other, researchers can uncover new strategies for enhancing the efficacy of biological control methods. This deeper comprehension could lead to more effective and targeted approaches in managing plant diseases, ultimately improving crop health and productivity.

## 5. Conclusions

In this study, we focused on evaluating the antagonistic activity of eight local *Trichoderma* spp. isolates, both in vitro and in vivo, against five isolates of *V. inaequalis*, the pathogen responsible for apple scab. The results indicated that *Trichoderma* spp. isolates were more effective when applied preventively rather than curatively, consistent with the performance of chemical fungicides. The observed variations in inhibition capacity between in vitro and in vivo conditions underscore the complex interactions between *Trichoderma* and the pathogen. The findings suggest that the effectiveness of *Trichoderma* spp. may be influenced by environmental factors, host plant conditions, and the specific mechanisms of action employed by different isolates. The in vitro tests suggested the presence of inhibitory substances in the filtrates, while the antifungal effects of volatile compounds produced by the strains were confirmed through in vitro antagonism tests. Of particular note, *T. virens* (Ol4) emerged as a promising candidate for the preventive control of apple scab, warranting further investigation into its application under field conditions. Future studies should focus on evaluating the long-term efficacy and stability of *Trichoderma* in field conditions, as well as its potential synergistic effects when used in combination with other biological control agents or cultural practices. By understanding the full potential of *Trichoderma* spp. in disease management, we can contribute to more sustainable agricultural practices and reduce reliance on chemical fungicides.

## Figures and Tables

**Table 1:** Inhibition rates of *V. inaequalis* mycelial growth observed with dual culture of *Trichoderma* spp. after 5 days of incubation at 25 °C.

	Pathogen Strains				
Treatment	ViAZ	ViIM	ViIF01	ViHA	ViEN01
Dif *	69.80 ± 1.99 ^a**^	73.00 ± 1.86 ^a^	69.37 ± 2.53 ^b^	80.23 ± 2.48 ^a^	71.64 ± 1.58 ^a^
G	51.67 ± 0.94 ^c^	45.57 ± 1.64 ^e^	38.50 ± 4.99 ^e^	43.09 ± 1.41 ^c^	50.05 ± 1.13 ^d^
Nef9	48.19 ± 0.99 ^d^	37.26 ± 1.26 ^j^	46.74 ± 2.20 ^d^	41.12 ± 2.85 ^c^	46.97 ± 2.05 ^d^
Ol4	58.04 ± 3.23 ^b^	56.60 ± 2.18 ^b^	73.82 ± 2.71 ^a^	61.35 ± 1.17 ^b^	66.97 ± 3.16 ^b^
T1	44.56 ± 2.05 ^e^	41.07 ± 1.92 ^f^	45.09 ± 2.90 ^d^	41.36 ± 0.95 ^c^	47.84 ± 2.19 ^d^
T2	41.15 ± 2.51 ^f^	44.73 ± 2.82 ^e^	46.40 ± 2.41 ^d^	39.75 ± 4.20 ^c^	48.63 ± 1.99 ^d^
Tricho	49.79 ± 2.17 ^cd^	48.38 ± 2.78 ^d^	48.86 ± 2.20 ^d^	44.76 ± 0.28 ^c^	57.46 ± 0.72 ^c^
X31	51.14 ± 0.99 ^c^	45.24 ± 1.60 ^e^	55.51 ± 0.62 ^c^	40.23 ± 4.36 ^c^	49.84 ± 0.90 ^d^
Y28	59.14 ± 0.93 ^b^	52.55 ± 2.09 ^c^	69.48 ± 2.91 ^b^	45.35 ± 1.99 ^c^	63.91 ± 1.12 ^b^

* Dif: difenoconazole fungicide; G, Nef9, …, Y28: *Trichoderma* isolate codes. ** In each column, values represent inhibition rates ± standard deviation. Data sharing the same letter are not significantly different according to the SNK test conducted on mycelial growth (*p* < 0.05).

**Table 2:** Inhibition rates of *V. inaequalis* mycelial growth observed with direct dual culture of *Trichoderma* spp. after 12 days of incubation at 25 °C.

	Pathogen Strains				
Treatment	ViAZ	ViIM	ViIF01	ViHA	ViEN01
Dif *	74.83 ± 2.27 ^a**^	77.81 ± 2.98 ^a^	76.41 ± 1.05 ^a^	73.03 ± 1.58 ^a^	75.63 ± 1.63 ^a^
G	70.10 ± 0.92 ^bc^	60.81 ± 4.73 ^b^	62.17 ± 3.29 ^c^	57.19 ± 0.96 ^c^	68.77 ± 0.54 ^b^
Nef9	66.35 ± 3.75 ^cde^	61.54 ± 3.17 ^b^	68.25 ± 0.52 ^bc^	59.19 ± 3.19 ^bc^	66.88 ± 3.49 ^b^
Ol4	68.66 ± 1.00 ^cd^	62.51 ± 1.29 ^b^	78.60 ± 5.42 ^a^	59.00 ± 1.10 ^bc^	64.73 ± 4.47 ^b^
T1	74.61 ± 1.78 ^a^	56.36 ± 5.20 ^bc^	68.60 ± 6.72 ^bc^	57.25 ± 2.84 ^c^	65.98 ± 3.20 ^b^
T2	66.53 ± 2.91 ^cde^	53.23 ± 5.14 ^c^	65.91 ± 1.34 ^bc^	61.09 ± 4.16 ^b^	64.43 ± 3.53 ^b^
Tricho	65.44 ± 4.33 ^de^	58.42 ± 2.35 ^bc^	70.08 ± 7.91 ^b^	61.38 ± 1.99 ^b^	50.19 ± 3.42 ^d^
X31	63.80 ± 2.40 ^e^	61.06 ± 2.01 ^b^	81.58 ± 3.59 ^a^	56.69 ± 0.68 ^c^	58.24 ± 1.11 ^c^
Y28	72.56 ± 0.63 ^ab^	59.18 ± 2.41 ^bc^	77.35 ± 4.94 ^a^	55.88 ± 0.92 ^c^	63.94 ± 1.84 ^b^

* Dif: difenoconazole fungicide; G, Nef9, …, Y28: *Trichoderma* isolate codes. ** In each column, values represent inhibition rates ±standard deviation. Data sharing the same letter are not significantly different according to the SNK test conducted on mycelial growth (*p* < 0.05).

**Table 3:** Inhibition rates of *V. inaequalis* mycelial growth caused by VOCs from *Trichoderma* spp. after 5 days of incubation at 25 °C.

	Pathogen Strains				
Treatments	ViAZ	ViIM	ViIF01	ViHA	ViEN01
G *	33.65 ± 9.59 ^b**^	7.46 ± 1.32 ^cd^	52.07 ± 0.80 ^ab^	15.21 ± 0.88 ^cd^	11.61 ± 9.49 ^c^
Nef9	30.02 ± 2.99 ^b^	21.11 ± 8.97 ^b^	16.25 ± 1.34 ^d^	39.53 ± 0.46 ^bc^	30.56 ± 1.00 ^b^
Ol4	34.65 ± 4.52 ^b^	18.26 ± 1.64 ^bc^	47.32 ± 6.33 ^b^	33.84 ± 4.21 ^bc^	27.17 ± 1.06 ^b^
T1	4.74 ± 2.13 ^c^	10.74 ± 0.96 ^bc^	40.92 ± 0.82 ^c^	13.79 ± 4.32 ^cd^	32.88 ± 0.49 ^b^
T2	29.91 ± 0.99 ^b^	16.89 ± 2.06 ^bc^	17.87 ± 1.11 ^d^	41.36 ± 37.47 ^bc^	24.80 ± 0.90 ^b^
Tricho	39.88 ± 9.06 ^b^	11.82 ± 6.28 ^bc^	58.41 ± 1.75 ^a^	77.92 ± 0.94 ^a^	54.05 ± 4.16 ^a^
X31	66.52 ± 3.68 ^a^	55.62 ± 5.28 ^a^	53.46 ± 7.22 ^ab^	60.82 ± 0.72 ^ab^	54.86 ± 0.74 ^a^
Y28	69.80 ± 1.63 ^a^	62.15 ± 4.18 ^a^	59.44 ± 0.79 ^a^	61.16 ± 3.67 ^ab^	61.26 ± 1.56 ^a^

* G, Nef9, …, Y28: *Trichoderma* isolate codes. ** In each column, values represent inhibition rates ± standard deviation. Data sharing the same letter are not significantly different according to the SNK test conducted on mycelial growth (*p* < 0.05).

**Table 4:** Inhibition rates of *V. inaequalis* mycelial growth caused by VOCs from *Trichoderma* spp. after 12 days of incubation at 25 °C.

	Pathogen Strains				
Treatments	ViAZ	ViIM	ViIF01	ViHA	ViEN01
G *	40.18 ± 2.28 ^d**^	15.58 ± 2.26 ^c^	12.98 ± 2.30 ^e^	35.14 ± 0.89 ^c^	35.86 ± 0.19 ^e^
Nef9	39.44 ± 1.55 ^d^	32.83 ± 11.20 ^b^	19.97 ± 1.70 ^cd^	59.37 ± 1.97 ^b^	48.22 ± 3.01 ^d^
Ol4	46.63 ± 1.25 ^c^	26.93 ± 1.03 ^b^	24.21 ± 5.79 ^c^	37.89 ± 2.51 ^c^	30.25 ± 0.56 ^f^
T1	5.65 ± 0.38 ^e^	17.13 ± 0.27 ^c^	11.61 ± 1.05 ^e^	14.01 ± 10.23 ^e^	26.02 ± 0.47 ^f^
T2	7.59 ± 1.47 ^e^	16.82 ± 3.65 ^c^	18.16 ± 0.29 ^d^	23.32 ± 1.33 ^d^	29.97 ± 0.74 ^f^
Tricho	45.24 ± 0.76 ^c^	16.31 ± 0.75 ^c^	65.99 ± 1.97 ^a^	77.84 ± 2.68 ^a^	58.65 ± 5.31 ^c^
X31	66.79 ± 0.82 ^b^	68.13 ± 1.67 ^a^	50.44 ± 1.01 ^b^	75.84 ± 1.28 ^a^	64.79 ± 1.66 ^b^
Y28	70.32 ± 1.91 ^a^	68.43 ± 2.06 ^a^	68.04 ± 0.26 ^a^	76.10 ± 0.99 ^a^	71.34 ± 0.39 ^a^

* G, Nef9, …, Y28: *Trichoderma* isolate codes. ** In each column, values represent inhibition rates ± standard deviation. Data sharing the same letter are not significantly different according to the SNK test conducted on mycelial growth (*p* < 0.05).

**Table 5:** Inhibition of *V. inaequalis* mycelial growth by free cell filtrates of antagonistic *Trichoderma* spp. after 5 days of incubation at 25 °C.

	Pathogen Strains				
Treatments	ViAZ	ViIM	ViIF01	ViHA	ViEN01
G *	52.31 ± 0.22 ^a**^	44.61 ± 0.78 ^b^	32.94 ± 1.54 ^d^	33.82 ± 1.12 ^d^	33.23 ± 1.90 ^e^
Nef9	47.15 ± 0.57 ^b^	41.09 ± 0.99 ^b^	58.42 ± 1.76 ^c^	58.70 ± 2.50 ^b^	50.16 ± 0.51 ^b^
Ol4	41.71 ± 1.57 ^c^	26.90 ± 1.76 ^c^	41.45 ± 1.16 ^d^	30.84 ± 2.39 ^d^	39.33 ± 2.38 ^d^
T1	52.77 ± 1.34 ^a^	14.36 ± 1.02 ^d^	72.60 ± 5.79 ^ab^	64.36 ± 2.54 ^a^	42.70 ± 1.86 ^c^
T2	33.74 ± 0.81 ^d^	14.83 ± 1.55 ^d^	39.66 ± 1.64 ^d^	31.44 ± 0.60 ^d^	28.49 ± 0.83 ^f^
Tricho	54.73 ± 1.10 ^a^	43.78 ± 1.06 ^b^	38.16 ± 1.53 ^d^	52.64 ± 1.38 ^c^	22.10 ± 1.40 ^j^
X31	35.86 ± 2.63 ^d^	28.81 ± 2.49 ^c^	66.87 ± 0.37 ^b^	24.00 ± 1.79 ^e^	15.94 ± 1.89 ^h^
Y28	16.25 ± 4.94 ^e^	63.41 ± 2.21 ^a^	78.71 ± 4.04 ^a^	57.92 ± 2.03 ^b^	64.20 ± 1.52 ^a^

* G, Nef9, …, Y28: *Trichoderma* isolate codes. ** In each column, values represent inhibition rates ± standard deviation. Data sharing the same letter are not significantly different according to the SNK test conducted on mycelial growth (*p* < 0.05).

**Table 6:** Inhibition of *V. inaequalis mycelial* growth by free cell filtrates of antagonistic *Trichoderma* spp. after 12 days of incubation at 25 °C.

	Pathogen Strains				
Treatments	ViAZ	ViIM	ViIF01	ViHA	ViEN01
G *	53.18 ± 0.25 ^abc**^	34.32 ± 0.53 ^c^	12.49 ± 2.32 ^f^	31.05 ± 0.80 ^e^	16.49 ± 5.17 ^e^
Nef9	42.41 ± 0.13 ^d^	25.11 ± 3.18 ^cd^	47.18 ± 2.77 ^c^	59.21 ± 0.18 ^b^	42.13 ± 0.88 ^c^
Ol4	49.37 ± 0.35 ^abcd^	61.36 ± 0.03 ^b^	50.15 ± 0.52 ^c^	44.77 ± 1.90 ^c^	62.39 ± 2.32 ^b^
T1	56.96 ± 1.70 ^a^	2.77 ± 5.55 ^e^	56.23 ± 0.63 ^b^	59.99 ± 0.68 ^b^	20.86 ± 0.44 ^e^
T2	49.67 ± 11.33 ^abcd^	10.90 ± 4.18 ^de^	28.73 ± 5.17 ^e^	24.79 ± 0.29 ^j^	26.41 ± 4.17 ^d^
Tricho	45.76 ± 0.73 ^bcd^	35.73 ± 0.68 ^c^	27.98 ± 0.17 ^e^	37.98 ± 0.97 ^d^	26.75 ± 0.94 ^d^
X31	55.18 ± 0.99 ^ab^	38.04 ± 7.20 ^c^	41.17 ± 2.06 ^d^	27.69 ± 1.07 ^f^	8.75 ± 1.38 ^f^
Y28	43.36 ± 0.65 ^cd^	78.33 ± 1.89 ^a^	78.45 ± 0.42 ^a^	63.68 ± 1.13 ^a^	79.08 ± 1.21 ^a^

* G, Nef9, …, Y28: *Trichoderma* isolate codes. ** In each column, values represent inhibition rates ± standard deviation. Data sharing the same letter are not significantly different according to the SNK test conducted on mycelial growth (*p* < 0.05).

**Table 7:** Mean severity of scab on detached leaves (in vivo) treated with *Trichoderma* spp. (Y28 and Ol4) and fungicide (Dif: difenoconazole).

	Pathogen Strains				
Treatment	ViAZ	ViEN01	ViHA	ViIF01	ViIM
Control ^X^	97.00 ± 1.63 ^a*^	98.25 ± 0.96 ^a^	98.50 ± 0.58 ^a^	97.50 ± 1.73 ^a^	96.75 ± 2.63 ^a^
Dif	41.25 ± 11.09 ^c^	45.75 ± 7.46 ^c^	48.25 ± 9.43 ^c^	70.75 ± 5.06 ^c^	57.00 ± 5.10 ^c^
Ol4	59.00 ± 8.68 ^b^	66.00 ± 14.85 ^b^	71.00 ± 4.90 ^b^	79.25 ± 2.06 ^b^	63.50 ± 1.73 ^b^
Y28	48.50 ± 8.27 ^bc^	62.00 ± 8.68 ^bc^	62.75 ± 5.32 ^c^	70.25 ± 10.24 ^b^	58.50 ± 3.70 ^b^
Control ^Y^	97.00 ± 1.63 ^a^	98.25 ± 0.96 ^a^	98.50 ± 0.58 ^a^	97.50 ± 1.73 ^a^	96.75 ± 2.63 ^a^
Dif	63.75 ± 8.54 ^c^	58.75 ± 4.79 ^c^	70.00 ± 4.08 ^c^	76.00 ± 3.46 ^c^	60.50 ± 9.04 ^c^
Ol4	74.00 ± 6.27 ^b^	61.50 ± 8.96 ^b^	80.00 ± 5.48 ^b^	83.50 ± 4.80 ^c^	81.00 ± 2.94 ^b^
Y28	70.25 ± 5.44 ^bc^	55.00 ± 7.48 ^b^	71.00 ± 5.72 ^c^	84.25 ± 6.29 ^b^	77.50 ± 7.72 ^b^
Control ^Z^	97.00 ± 1.63 ^a^	98.25 ± 0.96 ^a^	98.50 ± 0.58 ^a^	97.50 ± 1.73 ^a^	96.75 ± 2.63 ^a^
Dif	70.25 ± 10.66 ^b^	60.25 ± 4.57 ^c^	73.50 ± 3.87 ^c^	74.25 ± 3.77 ^b^	71.00 ± 6.22 ^c^
Ol4	78.75 ± 6.18 ^b^	81.25 ± 9.00 ^b^	76.75 ± 4.99 ^b^	85.00 ± 2.94 ^b^	80.25 ± 5.56 ^b^
Y28	78.25 ± 4.79 ^b^	74.75 ± 9.11 ^b^	74.50 ± 4.51 ^c^	74.50 ± 6.76 ^b^	80.50 ± 5.57 ^b^

* Values represent inhibition rates ± standard deviation. In each column, data with the same letter are not significantly different according to the SNK test (*p* < 0.05). ^X^: Preventive treatment, ^Y^: Curative treatment after 5 days, ^Z^: Curative treatment after symptom onset.

## Data Availability

The data that support the findings of this study are available from the corresponding author upon reasonable request.

## References

[1] El Jaouhari N., Abouabdillah A., Bouabid R., Bourioug M., Aleya L., Chaoui M. (2018). Assessment of Sustainable Deficit Irrigation in a Moroccan Apple Orchard as a Climate Change Adaptation Strategy. Sci. Total Environ..

[2] Moinina A., Lahlali R., Boulif M. (2019). Important Pests, Diseases and Weather Conditions Affecting Apple Production in Morocco: Current State and Perspectives. Rev. Marocaine Sci. Agron. Vétérinaires.

[3] Belete T., Boyraz N. (2017). Critical Review on Apple Scab (*Venturia inaequalis*) Biology, Epidemiology, Economic Importance, Management and Defense Mechanisms to the Causal Agent. J. Plant Physiol. Pathol..

[4] Shah A.A., Gupta A. (2023). *Venturia inaequalis* Post-Infection Enhancement of Secondary Metabolites in the Peels of Delicious Apple Variety. Mater. Today Proc..

[5] Stević M., Tamaš N., Miletić N., Vukša P. (2015). Different Toxicity of the Strobilurin Fungicides Kresoxim-Methyl and Trifloxistrobin to *Venturia inaequalis* Isolates from Serbia. J. Environ. Sci. Health Part B.

[6] Moinina A., Lahlali R., Maclean D., Boulif M. (2018). Farmers’ Knowledge, Perception and Practices in Apple Pest Management and Climate Change in the Fes-Meknes Region, Morocco. Horticulturae.

[7] Oukabli A. (2004). Le Pommier: Une Culture de Terroir En Zones d’altitude. Transf. Technol. Agric..

[8] Ezrari S., Legrifi I., Taoussi M., Khadiri M., Belabess Z., Lahlali R. (2023). Plant–Pathogen Interactions and Global Food Security. Plant Pathogen Interaction.

[9] Baker K.F. (1987). Evolving Concepts of Biological Control of Plant Pathogens. Annu. Rev. Phytopathol..

[10] Smith T., Taylor M.S. (1919). Some Morphological and Biological Characters of the Spirilla (Vibrio Fetus, n. Sp.) Associated with Disease of the Fetal Membranes in Cattle. J. Exp. Med..

[11] Köhl J., Kolnaar R., Ravensberg W.J. (2019). Mode of Action of Microbial Biological Control Agents Against Plant Diseases: Relevance Beyond Efficacy. Front. Plant Sci..

[12] Ghazanfar M.U., Raza M., Raza W., Qamar M.I. (2018). Trichoderma as Potential Biocontrol Agent, Its Exploitation in Agriculture: A Review. Plant Prot..

[13] Vinale F., Sivasithamparam K., Ghisalberti E.L., Ruocco M., Woo S., Lorito M. (2012). Trichoderma Secondary Metabolites That Affect Plant Metabolism. Nat. Prod. Commun..

[14] Muresan L.E. (2017). Cultivable Bacterial and Fungal Endophytes from Apple Tissues and Their Potential for Biological Control of *Venturia inaequalis*. Master’s Thesis.

[15] Omar I., O’neill T.M., Rossall S. (2006). Biological Control of Fusarium Crown and Root Rot of Tomato with Antagonistic Bacteria and Integrated Control When Combined with the Fungicide Carbendazim. Plant Pathol..

[16] Elshahawy I.E., El-mohamedy R.S. (2019). Biological Control of Pythium Damping-off and Root-Rot Diseases of Tomato Using Trichoderma Isolates Employed Alone or in Combination. J. Plant Pathol..

[17] Raut I., Badea-Doni M., Calin M., Oancea F., Vasilescu G., Sesan T.E., Jecu L. (2014). Effect of Volatile and Non-Volatile Metabolites from *Trichoderma* spp. against Important Phytopathogens. Rev. Chim..

[18] Boubaker H., Karim H., El Hamdaoui A., Msanda F., Leach D., Bombarda I., Vanloot P., Abbad A., Boudyach E.H., Aoumar A.A. (2016). Ben Chemical Characterization and Antifungal Activities of Four Thymus Species Essential Oils against Postharvest Fungal Pathogens of Citrus. Ind. Crops Prod..

[19] Nicholson R.L., Van Scoyoc S., Kuc J., Williams E.B. (1973). Response of Detached Apple Leaves to *Venturia inaequalis*. Phytopathology.

[20] Yepes L.M., Aldwinckle H.S. (1993). Selection of Resistance to *Venturia inaequalis* Using Detached Leaves from in Vitro-Grown Apple Shoots. Plant Sci..

[21] Calenge C., Maillard D., Fournier P., Fouque C. (2004). Efficiency of Spreading Maize in the Garrigues to Reduce Wild Boar (Sus Scrofa) Damage to Mediterranean Vineyards. Eur. J. Wildl. Res..

[22] Gusella G., Vitale A., Polizzi G. (2022). Potential Role of Biocontrol Agents for Sustainable Management of Fungal Pathogens Causing Canker and Fruit Rot of Pistachio in Italy. Pathogens.

[23] Rai S., Agrawal C., Shrivastava A.K., Singh P.K., Rai L.C. (2014). Comparative Proteomics Unveils Cross Species Variations in Anabaena under Salt Stress. J. Proteom..

[24] Terhonen E., Blumenstein K., Kovalchuk A., Asiegbu F.O. (2019). Forest Tree Microbiomes and Associated Fungal Endophytes: Functional Roles and Impact on Forest Health. Forests.

[25] Belabess Z., Gajjout B., Legrifi I., Barka E.A., Lahlali R. (2024). Exploring the Antifungal Activity of Moroccan Bacterial and Fungal Isolates and a Strobilurin Fungicide in the Control of Cladosporium Fulvum, the Causal Agent of Tomato Leaf. Plants.

[26] Nourian A., Salehi M., Safaie N., Khelghatibana F. (2024). Biocontrol of Diplodia Bulgarica, the Causal Agent of Apple Canker, Using Trichoderma Zelobreve. Arch. Microbiol..

[27] Taha M., Mostafa A.A., Al-askar A.A., Sayed S.R.M., Mostafa A. (2021). Antagonistic Activity of Trichoderma Harzianum and Trichoderma Viride Strains against Some Fusarial Pathogens Causing Stalk Rot Disease of Maize, in Vitro. J. King Saud Univ.-Sci..

[28] Singh J., Kumar V., Srivastava S., Kumar A., Singh V.P. (2018). In Vitro Evaluation of Trichoderma Species against Fusarium Oxysporum f. Sp. Lycopersici Causing Tomato Wilt. Plant Pathol. J..

[29] Golafrouz H., Safaie N., Khelghatibana F. (2020). The Reaction of Some Apple Rootstocks to Biocontrol of White Root Rot Rosellinia Necatrix by Trichoderma Harzianum in Greenhouse. J. Crop Prot..

[30] Jimenez M., Hernández F.D., Alcalá E.I.L., Morales G.G., Valdés R.A., Reyes F.C. (2018). Biological Effectiveness of Bacillus Spp. and *Trichoderma* spp. on Apple Scab (*Venturia inaequalis*) in Vitro and under Field Conditions. Eur. J. Phys. Agric. Sci..

[31] Doolotkeldieva T., Bobusheva S. (2017). Scab Disease Caused by *Venturia inaequalis* on Apple Trees in Kyrgyzstan and Biological Agents to Control This Disease. Adv. Microbiol..

[32] Kumar N., Khurana S.M.P. (2021). Trichoderma-Plant-Pathogen Interactions for Benefit of Agriculture and Environment. Biocontrol Agents and Secondary Metabolites.

[33] Phoka N., Suwannarach N., Lumyong S., Ito S.I., Matsui K., Arikit S., Sunpapao A. (2020). Role of Volatiles from the Endophytic Fungus Trichoderma Asperelloides PSU-P1 in Biocontrol Potential and in Promoting the Plant Growth of Arabidopsis Thaliana. J. Fungi.

[34] Bhale U.N., Rajkonda J.N. (2012). Evaluation of Distribution of Trichoderma Species in Soils of Marathwada Region of Maharashtra during 2007–2011. J. Mycol. Plant Pathol..

[35] Köhl J., Scheer C., Holb I.J., Masny S., Molhoek W. (2015). Toward an Integrated Use of Biological Control by Cladosporium Cladosporioides H39 in Apple Scab (*Venturia inaequalis*) Management. Plant Dis..

[36] Harman G.E. (2006). Overview of Mechanisms and Uses of *Trichoderma* spp.. Phytopathology.

[37] Moragrega C., Carmona A., Llorente I. (2021). Biocontrol of Stemphylium Vesicarium and Pleospora Allii on Pear by Bacillus Subtilis and *Trichoderma* spp.: Preventative and Curative Effects on Inoculum Production. Agronomy.

[38] Rao Y., Zeng L., Jiang H., Mei L., Wang Y. (2022). Trichoderma Atroviride LZ42 Releases Volatile Organic Compounds Promoting Plant Growth and Suppressing Fusarium Wilt Disease in Tomato Seedlings. BMC Microbiol..

[39] Jeyaseelan E.C., Tharmila S., Niranjan K. (2012). Antagonistic Activity of *Trichoderma* spp. and Bacillus Spp. against Pythium Aphanidermatum Isolated from Tomato Damping Off. Arch. Appl. Sci. Res..

[40] Yu Z.-F., Qiao M., Zhang Y., Zhang K.-Q. (2007). Two New Species of Trichoderma from Yunnan, China. Antonie Van Leeuwenhoek.

[41] Strobel G. (2018). The Emergence of Endophytic Microbes and Their Biological Promise. J. Fungi.

[42] Speckbacher V., Ruzsanyi V., Wigger M., Zeilinger S. (2020). The Trichoderma Atroviride Strains P1 and IMI 206040 Differ in Their Light-Response and VOC Production. Molecules.

[43] Atanasova L., Le Crom S., Gruber S., Coulpier F., Seidl-Seiboth V., Kubicek C.P., Druzhinina I.S. (2013). Comparative Transcriptomics Reveals Different Strategies of Trichoderma Mycoparasitism. BMC Genom..

[44] Zeilinger S., Gruber S., Bansal R., Mukherjee P.K. (2016). Secondary Metabolism in Trichoderma–Chemistry Meets Genomics. Fungal Biol. Rev..

[45] Sood M., Kapoor D., Kumar V., Sheteiwy M.S. (2020). Trichoderma: The “Secrets” of a Multitalented. Plants.

